# Metabolic Power of Female Footballers in Various Small-Sided Games with Different Pitch Surfaces and Sizes

**DOI:** 10.3390/sports5020024

**Published:** 2017-04-17

**Authors:** Jorge López-Fernández, Javier Sánchez-Sánchez, Leonor Gallardo, Jorge García-Unanue

**Affiliations:** 1 IGOID Research Group, University of Castilla-La Mancha, 45071 Toledo, Spain; leonor.gallardo@uclm.es; 2 School of Sport Science, European University, 28670 Villaviciosa de Odón, Spain; javier.sanchez2@universidadeuropea.es (J.S.-S.); jorge.garcia2@universidadeuropea.es (J.G.-U.)

**Keywords:** artificial turf, four-a-side games, GPS, soccer, sports pavement

## Abstract

Small-sided-games (SSGs) seem to be a useful tool for replicating most types of scenarios found in sport competitions, but it is not that clear in female soccer. Game surface and pitch size seem to affect the intensity of SSGs, but no one has yet analysed the influence of these two variables together. The objective of this research was to analyse the metabolic power demands of various SSGs on possession play without goal-keepers, played on three different surfaces. Sixteen sub-elite female players performed three different four-a-side games (400 m^2^, 600 m^2^, and 800 m^2^) on three different surfaces (ground [GR]; natural grass [NG]; and artificial turf [AT]), recording a total of 96 events. Metabolic variables were recorded through a global positioning system (GPS). The GR condition obtained the lowest outputs for all variables in all of the SSGs. Furthermore, NG resulted in higher outcomes than AT for Average Metabolic Power (SSG 400 [+0.65; *p* = 0.019]; SSG 600 [+0.70; *p* = 0.04]); and equivalent distance (SSG 400 [+33.0; *p* = 0.02]; SSG 600 [+36.53; *p* = 0.04]). Moreover, SSG 400 obtained lower results than SSG 600 and SSG 800 for both AT and NG. In conclusion, playing on GR reduces the metabolic power of SSGs, While NG seems to be the most suitable surface for attaining highest metabolic responses for sub-elite female players. On the other hand, too big a pitch size may not increase the metabolic demands of the game.

## 1. Introduction

While the physical performance of female footballers is still a growing area of research [1], there are now a number of studies that quantify the physical performance of females during matches [2,3,4,5,6,7]. Like male footballers, high-intensity actions in female football are considered the most relevant in performance in spite of their short duration, as they are most often actions in goal situations [7,8]. Nevertheless, high-intensity actions are related to an increase in metabolic demands [9], causing muscle break-down, oxidative stress, and both biochemical and hormonal variations as a result of the eccentric component of these actions [3,9,10]. 

To quantify the effect of high-intensity actions either in matches and training, several instruments like the Global Positioning System (GPS) are becoming popular [11,12,13]. Indeed, several studies have focussed on quantifying these actions in small-sided games (SSGs), as coaches have been using such games for years to replicate competition demands [14,15,16]. Nevertheless, to the authors’ knowledge, only Gabbett and Mulvey [2] and Mara et al. [14] have focused their research on SSGs in the context of female footballers, suggesting that SSGs are a useful tool for replicating aerobic and movement patterns, but that they may not provide sufficient high-intensity or repeated sprint stimuli. Traditionally, researchers have used both speed actions and distances to assess the body load of both training and matches. However, nowadays some authors are recommending the use of metabolic power and energy costs to assess the intensity in football, as these variables also account for accelerations at high intensity [17,18,19]. 

According to Di Prampero et al. [20], and later Osngnach et al. [21], all accelerations performed on a flat surface are equivalent to running on a slope the gradient of which is established by this acceleration. Therefore, through GPS devices and other tracking instruments, it is possible to estimate the metabolic power of a task. In spite of the controversy concerning the use of GPS devices for this purpose [22], several authors have reported that high-speed actions seem to underestimate the real load of a task regarding metabolic power [18,19,21]. Indeed, Gaudino, Alberti, and Iaia [17] reported greater high-intensity distance runs at high metabolic load (>20 W/kg) than at high speed (>14.4 km·h^−1^) when they studied the intensity of different SSGs with goalkeepers. Moreover, this argument may be confirmed by the findings of Akenhead et al. [23], as they found that 18% of the total distance covered is due to accelerations or decelerations at intensities greater than 1 m^2^/s. Therefore, it seems that coaches should use the metabolic variables to compile more accurate information on the demands of training [17,24]. 

Previous studies on SSGs demonstrated that external factors, such as the number of players or touches, the length of the game, the pitch size, and the game surface cause different physical and physiological responses, and therefore affect the duration and number of high-intensity actions [12,16,25,26,27]. However, there is still a lack of knowledge about how physiological responses can change when two or more of these variables are combined. Among all of these variables, several authors consider the game surface especially important, due to it being part of the interaction between player and pitch [13,28,29], as high-intensity responses are related to their mechanical properties [13,25], so that surfaces with lower damping capacities, such as sand, reduce high-intensity actions in SSGs [25]. However, these studies only assessed the high-intensity activities through the traditional way, not by providing information about the metabolic power variables. To the authors’ knowledge, only Gaudino et al. [17] have investigated the metabolic power responses of footballers on different surfaces, but using a standardised test instead of SSGs. Nonetheless, their findings confirm that the metabolic demands of high-intensity games are also altered by the surface. On the other hand, pitch size is also considered a key factor by authors such as Fradua et al. [30], given that players’ abilities to play soccer in small spaces is an essential element in this sport. The evidence from SSGs demonstrates that football players perform a higher number of high-intensity actions whenever the pitch size increases [11,31]. However, no authors have studied the influence of this variable in women footballers or its effect on metabolic responses. 

To address the gap in the literature on the effect of two or more extrinsic factors on SSG performance, the current research aims to analyse the metabolic power demands of various SSGs on possession play without goal-keepers, played on three different surfaces. As players seem to change some technical and tactical parameters according to the surface they are playing on, and players’ load is also affected [25,32], the intensity of SSGs with different pitch sizes may also be influenced by the game surface. Finally, we focused on possession because previous research suggests that this format increases the game intensity in comparison to SSGs with goalkeepers [17]. Therefore, the authors expect that the results of this study will help to provide relevant information for designing training based on the use of SSGs. 

## 2. Methods 

### 2.1. Participants

Based on a convenience sample approach, sixteen women belonging to a Spanish Second Division team took part in this study (19.56 ± 1.97 year; 57.74 ± 4.89 kg; 161.57 ± 5.83 cm; 24.93% ± 4.1% body fat). They had previous experience playing and training on both natural grass and artificial turf (5.81 ± 0.75 year). They also played about four to five matches on the ground every season, although most of them were friendly fixtures during the pre-season. All participants played football three times per week with a weekly match. As a requirement to participate in the study, players had to present the medical certificate required to play football. They testified that they were not taking any medication during the study and were free of cardiopulmonary diseases.

Both coaches and footballers signed the informed consent form testifying that they understood the possible risks of this investigation. Furthermore, the methodology of this research was approved by the local Clinical Research Ethical Committee in accordance with the Declaration of Helsinki.

### 2.2. Experimental Design

Prior to starting the study, the footballers took part in a familiarisation session to become accustomed to the Global Positioning System (GPS; Spi Pro X, GPSports, Canberra, Australia) and gain experience with both the three SSG pitch sizes and the three surfaces included in the study. The main part of this research was divided into three consecutive weeks (2 days per week) so that footballers played three four-a-side games with different pitch sizes (Table 1) on three different surfaces: ground (GR; uniform and dry dirt), natural grass (NG; height of grass: 25 mm), and artificial turf (AT; fibre: monofilament of polyethylene of 60 mm in height; infill: 20 kg∙m^−2^ of styrene-butadiene rubber (SBR) and quartz sand with 0.3–0.8 granulometry). To increase the reliability of data, players played each SSG on each surface twice, playing in a total 18 matches. Every test-day, participants played three SSGs, one per surface. The order of both the pitch sizes and the surfaces were established randomly for each test-day, ultimately recording a total of 96 events. To guarantee a full recovery between SSGs, 10-min periods of active rest were performed by footballers (ball pass exercises at low intensity together with three incremental sprints at the end of the recovery time). Tests were performed during regular training times (19:00–21:00) to avoid the influence of circadian rhythms and were conducted under similar weather conditions (dry, 20–24.5 °C and 22%–30% relative humidity). Finally, the mechanical properties of the surfaces were not measured in this study. However, an independent expert on sports ground surfaces stated that the three surfaces were in good condition for playing football.

### 2.3. Experimental Protocol

The participants agreed not to perform either vigorous or exhausting physical activity before each test. In addition, they used the same football boots (always rubber studs) and maintained the same eating habits. Both the SSGs and surfaces were established randomly.

The GPS devices were attached to players 15 min before the beginning of the tests. They used the same device during the whole investigation to guarantee the reliability of data. Contact with a minimum of eight satellites was established to guarantee the accuracy of data [17]. Subsequently, footballers carried out a standardised warm-up for 10 min and three sprints of 30 m at increasing intensity [13,29].

*Four-a-side game*: The coaches gathered the footballers in four teams based on their skill levels to guarantee equitable teams. Teams and matches were held the same during the investigation and coaches encouraged the players the entire time. To optimise the playing time, balls were replaced when they went outside the pitch. Contrary to previous research, the objective of all SSGs was possession without goal-keepers; therefore, neither goalkeepers nor goals were included in this study [11,25,31]. We included only SSGs with possession because they seem to increase the intensity of games in comparison to those with goal-keepers [17].

*Metabolic power*: The variables of metabolic power were calculated through the manufacturer software Team AMS (version 2016.7, GPSports, Canberra, Australia) following the methodology and equations used by Di Prampero et al. [20] and Osgnach et al. [21]. Thus, the GPS devices were used to register the Metabolic Load Absolute (KJ); Metabolic Load Relative (KJ/kg); the Average Metabolic Power (rate of energy consumed per second) (W/kg); High Metabolic Load Distance (total distance covered at 20 W/kg or more) (m); and the Equivalent Distance (maximum distance that the footballer could have run with the total energy consumed if she ran at constant speed) (m). Following the methodology of Gaudino et al. [17,33], the GPS devices recorded at 15 Hz (5 Hz GPS unit interpolated to 15 Hz) [34,35]. Moreover, this study used the same GPS model as in previous research to increase data reliability.

### 2.4. Data Analysis

Data were analysed through the statistical software SPSS v 20.0 (IBM, Armonk, NY, USA). The level of significance was established at *p* < 0.05. The results are presented as means and standard deviations (±SD). The verification of the normality and homogeneity of the variance was assumed by means of the Kolmogorov-Smirnov test and Levene’s statistic. A two-way repeated measures linear mixed model (surface x pitch size) was used to compare results among SSGs, whereas the interactions were identified through Bonferroni post-hoc pairwise comparisons. Effect sizes (ES) were calculated and defined as follows: trivial <0.19; small, 0.2–0.49; medium 0.5–0.79; large >0.8 [36]. 

## 3. Results

Table 2 displays the results of the metabolic variables. Footballers obtained lower values on GR than NG in the three SSGs (SSG 400, SSG 600, and SSG 800) for all metabolic variables. Moreover, GR presented lower outputs than AT for all metabolic variables, but only in SSG 600 and SSG 800. The results also show higher outcomes on NG than AT for the variables of Metabolic Load Relative (SSG 400 [+0.16; *p* = 0.017; ES: 0.762; CI: 0.02–1.29]); Metabolic Power (SSG 400 [+0.65; *p* = 0.019; ES: 0.749; CI: 0.08–1.21]; SSG 600 [+0.70; *p* = 0.04; ES: 0.648; CI: 0.02–1.38]); and Equivalent Distance (SSG 400 [+33.0; *p* = 0.02; ES: 0.738; CI: 4.18–61.82]; SSG 600 [+36.53; *p* = 0.04; ES: 0.662; CI: 1.21–71.84]). On the other hand, SSG 400 obtained lower results than SSG 600 and SSG 800 for all metabolic variables related to metabolic power in both the AT and NG conditions.

## 4. Discussion

The main objective of this research was to analyse and determine the effect of altering both pitch size and the game surface on the metabolic power of sub-elite female footballers in SSGs using a four-a-side format. However, contrary to previous studies, the objective was focused on possession games [11,25,31] because this sort of SSG seems to result in higher intensity levels than those that include goal-keepers [17]. The main results show that metabolic load is influenced by both the game surface and the pitch size; thus, in line with Gaudino, Alberti, and Iaia [17], different SSG formats were shown to have effects on different performance indicators. However, the lower outcomes in Metabolic Load Relative and Average Metabolic Power in our study than those obtained by Gaudino et al. [17] suggest that SSGs in sub-elite female football are less intense than in elite male football; therefore, comparisons between studies should be done with caution. Moreover, it is important to be cautious when extrapolating these findings to another sort of SSG, as they may be different in other formats such as three-a-side or five-a-side.

The significant differences among surfaces indicate that the metabolic demands change according to the game surface characteristics, which is in line with the findings of Brito et al. [25], although they assessed high-intensity actions on turf, asphalt, and sand. Among the surfaces analysed in this study, GR seems to be the less recommended surface for high-intensity actions, as the energy costs—both relative (Metabolic Load Relative) and absolute (Metabolic Load Absolute)—to complete the SSGs played on GR were lower than on NG and AT. Moreover, players’ work rate was lower on GR than on the other two surfaces, as the rates of energy expended per second (Average Metabolic Power) observed in the participants were also inferior on GR. These outcomes may be due to such a high rotational traction of GR affecting the players’ stability [13,29], thereby causing a lower number of explosive actions [9,25]. On the other hand, the higher Metabolic Power and Equivalent Distance on NG than on AT, both for SSG 400 and SSG 600, suggest a higher rate of creatine phosphate breakdown and glycolysis on NG as a consequence of greater rates of anaerobic energy turnover [9,13,25], although no differences were found between AT and NG for SSG 800. These findings contradict the conclusions of previous studies in men, as they found that the newest artificial turf systems do not entail lower performance in linear sprint nor cause greater fatigue [37]. However, this may be due to different technical behaviour on both surfaces, since players perform a higher number of short passes and a lower rate of tackles on AT than on NG [32]. Nonetheless, the high variability existing in the mechanical properties of artificial turf systems makes further research necessary [29].

Previous research in men footballers concluded that bigger pitches improve the intensity of the game in male footballer so that, variables such as sprint rate, distance covered at high-intensity speed, workload or work-rest rate increase in SSGs with a higher pitch ratio per player [11,31]. The findings of our research are in line with those previous studies, as SSG 400 resulted in lower values in all metabolic variables in comparison to the other two bigger pitches. In addition, the lower rate of energy expended per second (Average Metabolic Power) for SSG 400 indicates a higher intermittent activity of players in this SSG [11]. Therefore, the higher effective playing time associated with bigger pitches could explain the lower values of Average Metabolic Power and the lower outcomes in metabolic demands of these situations [11]. On the other hand, it should be noted that a few studies with SSGs did not report this trend with increases in individual playing area [27]. In line with these other studies, this research found that metabolic responses stop to increase if the pitch size of the SSG is too big, as it is supported by the lack of differences between the SSG 600 and SSG 800 sub-elite female players. Therefore, similar muscle damage, oxidative stress, and biochemical and hormonal variations in female players [3] may be expected for either SSG 600 or SSG. These findings are important, as either technical or tactical behaviour of players seem to also be different according to the pitch size [27]. Thus, coaches should not overlook pitch size when they use it as a control variable for the intensity of the game, as there is no need to increase the pitch size in excess. 

This research, therefore, will help coaches to design SSGs and training with greater accuracy, as both the game surface and pitch size seem to influence the high-intensity demand of one task. Nonetheless, it is necessary to be cautious when interpreting these findings due to the short duration of the SSGs used in this study and the lack of previous investigations of SSGs and metabolic power in female footballers. Moreover, contrary to previous research, the objective of the SSGs used in this research was possession, which seems to result in higher intensity levels in comparison to SSGs that include goal-keepers [11,17,25,31]. Therefore, more investigations are required to establish the relationship between these two variables with the intensity of the game. 

Finally, despite the increasing use of these variables in studies investigating football teams, some doubts have been cast about the reliability of GPS systems in measuring metabolic load [19,21,38]. Indeed, Buchheit et al. [22] found that data recorded through GPS systems considerably underestimate the energetic demands of a task, especially during rest phases. However, Coutts et al. [38] concluded that metabolic power variables may contribute to improving our understanding of the physical demands of collective sports. The main concern about using GPS devices for measuring the Metabolic Power variables is in regard to their reliability in measuring accelerations, decelerations, and high-speed actions accurately. For that reason, there is a need to exercise caution when interpreting the findings of this manuscript. Nevertheless, according to Osgnach et al. [39], GPS systems that record at 10Hz or more may be considered valid for measuring Metabolic Load, although there are other variables that can affect the quality of data.

## 5. Conclusions

This study focussed on possession games played by sub-elite women footballers. Nevertheless, the findings are in line with previous studies in men, as the game intensity levels of small-sided games were altered by both game surface and pitch size. 

Regarding the game surface, outcomes of this work evidence that playing football on ground reduces the intensity of the tasks. Thus, coaches should avoid this surface for training when they want players to work at the highest intensity possible. The differences found between natural grass and artificial turf show higher metabolic demands than natural surfaces. Therefore, contrary to the findings in male football, higher game intensity on natural grass than on turf in sub-elite female football may be expected. 

On the other hand, pitch size also influences the metabolic demands of small-sided games. Consequently, coaches should not overlook this variable when designing training drills, as women seem to play more intensely on bigger pitches. Nonetheless, the findings of this research evidence that metabolic responses stop increasing when the pitch size is too big. Therefore, whenever trainers use big pitches in small-sided games, they should aim to choose the size that better fits additional objectives, like improving players’ tactical or technical skills.

## Figures and Tables

**Table 1:** Small-sided game (SSG) characteristics.

Name of SSG *	Game Objective	Game Duration (min)	Pitch Area (m)	Pitch Total Area (m^2^)	Pitch Ratio Per Player (m^2^)
SSG 400	Possession game without goal-keepers	4	20 × 20 m	400 m^2^	50 m^2^
SSG 600		4	24.5 × 24.5 m	600 m^2^	75 m^2^
SSG 800		4	28.3 × 28.3 m	800 m^2^	100 m^2^

SSG: Small-Sided Game. * Footballers replayed each SSG in a non-consecutive test-day to increase data reliability.

**Table 2:** Metabolic Load Parameters on the three surfaces and the three SSG pitch sizes.

Metabolic Power’s Variables	Natural Grass (NG) (*)			Artificial Turf (AT) (^#^)			Ground (GR) (^†^)		
	SGG 400 (^a^)	SSG 600 (^b^)	SSG 800 (^c^)	SGG 400 (^a^)	SSG 600 (^b^)	SSG 800 (^c^)	SGG 400 (^a^)	SSG 600 (^b^)	SSG 800 (^c^)
Metabolic Load Relative (KJ/kg)	2.26 (0.19) ^†,#^	2.57 (0.28) ^†,a^	2.56 (0.29) ^†,a^	2.10 (0.23)	2.40 (0.26) ^†,a^	2.47 (0.21) ^†,a^	2.04 (0.25)	2.17 (0.34)	2.30 (0.23) ^a^
Average Metabolic Power (W/kg)	9.38 (0.82) ^†,#^	10.67 (1.12) ^#,†,a^	10.66 (1.21) ^†,a^	8.73 (0.93)	9.97 (1.04) ^†,a^	10.31 (0.87) ^†,a^	8.48 (1.04)	9.02 (1.40)	9.59 (0.96) ^a^
High Metabolic Load Distance (m)	65.39 (15.80) ^†^	89.48 (26.72) ^†,a^	99.14 (28.48) ^†,a^	56.27 (15.07)	77.35 (25.00) ^†,a^	88.94 (18.96) ^†,a^	50.53 (19.48)	57.56 (32.56)	70.88 (17.00) ^a^
Equivalent Distance (m)	484.82 (42.25) ^†,#^	552.21 (57.63) ^#,†,a^	551.89 (62.80) ^†,a^	451.82 (47.22)	515.68 (54.26) ^†,a^	532.94 (44.68) ^†,a^	438.69 (54.11)	466.34 (72.57)	496.66 (49.26) ^a^
Metabolic Load Absolute (KJ)	132.57 (19.40) ^†^	148.95 (19.80) ^†,a^	149.82 (19.77) ^†,a^	121.44 (20.29)	138.86 (17.24) ^†,a^	144.90 (17.97) ^a^	115.28 (18.04)	124.61 (20.36)	134.77 (15.55) ^a^

^*,#,†^ Significant differences with the surface indicated (*p* < 0.05). ^a,b,c^ Significant differences with the size indicated (*p* < 0.05). NG = Natural Grass; AT = Artificial Turf; GR = Ground; SSG400 = small-sided game with 400 m^2^ playing area; SSG 600 = small-sided game with 600 m^2^ playing area; SSG 800 = small-sided game with 800 m^2^ playing area.
